# Dietary Copper Intake and Bone Health: A Systematic Review and Meta-Analysis of Observational Studies

**DOI:** 10.1007/s00223-025-01463-w

**Published:** 2025-12-09

**Authors:** María Auxiliadora Gutiérrez-Guerra, Luis Manuel Puerto-Parejo, Elena Pastor-Ramón, María Pedrera-Canal, Vicente Vera, Juan Diego Pedrera-Zamorano, Jesús María Lavado-García, Fidel López-Espuela, Raúl Roncero-Martín, Juan Fabregat-Fernández, Jose M. Morán

**Affiliations:** 1 Socio-Health Sciences Research, University of Extremadura, Cáceres, Spain; 2 Gerencia del Área de Salud de Badajoz, Supervisor del Área de Investigación, Proyectos y Gestión, Av. de Huelva, 8, 06005 Badajoz, Spain; 3 Biblioteca Virtual de ciencias de la Salud de las Illes Balears (Bibliosalut), Ctra. De Valldemossa, 79, mòdul L+1, 07120 Palma, Spain; 4 Hospital Clínico San Carlos, Complutense University of Madrid, Madrid, Spain; 5 Department of Conservative Dentistry and Prostheses, Faculty of Dentistry, Complutense University of Madrid, Madrid, Spain; 6 Facultad de Enfermería y Terapia Ocupacional,Departamento de Enfermería, Universidad de Extremadura, 10001 Cáceres, Spain

**Keywords:** Dietary copper intake, Bone mineral density, Meta-analysis, Osteoporosis prevention

## Abstract

Studies evaluating habitual dietary copper intake and bone mineral density have garnered significant interest due to copper’s indispensable role in collagen cross-linking and osteogenesis. These investigations, which employ dietary assessment tools alongside DXA measurements of skeletal sites, have nonetheless yielded heterogeneous results regarding the impact of copper consumption on bone health. Consequently, elucidating the nature and magnitude of this association is of paramount importance for both nutritional epidemiology and osteoporosis prevention. The review was conducted in accordance with PRISMA guidelines and registered in Prospero (CRD42024617075). Electronic literature searches were performed up to February 2025 in EMBASE, PubMed, OVID, Scopus, and Web of Science to identify observational studies assessing dietary copper intake and DXA-measured BMD, and study quality was appraised using the Newcastle–Ottawa Scale. Data were pooled via a generic inverse-variance random-effects model, with heterogeneity assessed by the Q test and I^2^ statistic. A random-effects meta‐analysis of three studies (n = 9059) found that higher dietary copper intake was associated with a modest but significant increase in lumbar spine BMD (MD 0.02 g/cm^2^; 95% CI 0.00–0.04; *p* = 0.04; I^2^ = 36%), whereas a separate meta‐analysis of four studies (n = 14,345) for hip BMD showed a similar MD of 0.02 g/cm^2^ that did not reach significance (95% CI − 0.00–0.04; *p* = 0.07; I^2^ = 74%). Higher dietary copper intake is modestly associated with increased lumbar spine BMD, while evidence for hip BMD remains inconclusive, underscoring copper’s potential role in osteoporosis prevention.

**Supplementary Information:**

The online version contains supplementary material available at 10.1007/s00223-025-01463-w.

## Introduction

Dietary components are recognized as important environmental factors that can affect musculoskeletal diseases [1–7]. Trace elements, in addition to well-established risk factors such as age and BMI, are receiving an increased emphasis for their role in the development of osteoporosis [8–12]. Copper is an essential trace element that is vital for sustaining human health [3, 13–17] and is specifically necessary for the proper growth, development, and maintenance of bone tissue [4, 5, 7, 18, 19]. Unlike some other nutrients, copper is a trace mineral that cannot be synthesized in the human body and must be ingested solely from dietary sources [14, 17]. Given the crucial role of copper in processes essential for bone structure, such as collagen cross-linking [20–23] and the promotion of osteogenesis [24, 25], understanding how dietary intake levels are related to bone health outcomes is important.

However, the relationship between copper status and the risk of osteoporosis has rarely been studied in observational studies, and the relationship between copper intake and bone mineral density (BMD) has not been extensively evaluated in the general adult population until recently [5, 7, 14, 19, 26]. Previous research has often focused on the association between serum copper levels and bone health, which has yielded mixed results [5, 7, 19, 26, 27]. These studies, while providing some insights, did not explore the direct relationship between copper intake and bone health conditions such as osteoporosis.

Studies that specifically assess dietary copper intake often use tools such as 24-hour recalls or food frequency questionnaires [14, 28, 29]. Despite the value of dietary studies, methods such as dietary recall questionnaires have limitations, such as potential recall bias and inaccurate capture of long-term dietary habits [28, 30]. However, they remain useful tools for investigating nutrient intake in relation to health outcomes [15, 31].

The use of quantitative techniques is crucial for determining bone health. Among these techniques, dual-energy X-ray absorptiometry (DXA) is a primary method. DXA is recognized as the gold standard for the diagnosis of osteoporosis worldwide [32]. DXA measures BMD. DXA assessments provide accurate BMD determinations in critical skeletal regions important for diagnosis, such as the lumbar spine (L2–L4) and the femur (including the whole femur, femoral neck, and trochanter), allowing for the classification of individuals into categories of normal bone mass, osteopenia, and osteoporosis on the basis of T scores or standard deviations below the young adult reference [33]. In research, particularly in dietary studies, DXA is extensively used to evaluate the association between nutrient intake and bone health outcomes; particularly, studies use DXA measurements of BMD at various sites to investigate how factors such as dietary copper intake are related to bone density or the risk of osteoporosis [7, 14, 30]. BMD is crucial in the assessment of osteoporosis, a systemic skeletal disease primarily characterized by markedly deficient BMD and consequently excessive susceptibility to fracture [34]. A low BMD is directly associated with an increased risk of fracture [35]. Therefore, quantifying BMD is fundamental for evaluating bone health status and diagnosing conditions such as osteoporosis and osteopenia.

Osteoporosis remains a leading cause of fracture-related morbidity worldwide, yet the role of trace minerals in its pathogenesis is still incompletely defined. Copper is biologically poised to influence skeletal integrity. However, population evidence is inconclusive: large cross-sectional surveys such as the NHANES have linked higher copper intake to greater BMD, whereas other cohort and case‑control studies (particularly in postmenopausal women) have reported no association or even inverse associations [6, 14, 28]. These discrepancies stem from the predominantly observational nature of the data, variability in dietary assessment methods, and heterogeneity in the study populations.

Because most of the available studies quantify bone health status objectively with DXA, the evidence is amenable to rigorous quantitative synthesis. We therefore undertook a systematic review and meta‑analysis of observational studies that measured habitual dietary copper intake and DXA‑derived bone health outcomes. Our goals are to (i) generate the first pooled estimate of the copper–bone association, (ii) explore sources of between‑study heterogeneity, and (iii) assess whether the magnitude of any observed effect warrants the inclusion of copper in dietary guidance or targeted interventions for osteoporosis prevention. By providing a transparent, data-driven appraisal of the evidence (and explicitly accounting for conflicting findings), this study seeks to inform both clinical decision-making and public health nutrition policy.

## Materials and Methods

The review was conducted following the indications of the Preferred Reporting Items for Systematic Reviews and Meta-Analyses (PRISMA) statement [36] (Supplementary Material S1) and was also registered (Prospero database: CRD42024617075).

### Search Strategy

Three researchers (JMM, LMP-P and EP-R) each carried out an electronic literature search in the EMBASE, PubMed, OVID, Scopus and Web of Science databases. No language restrictions were imposed, and all publications up to February 2025 were considered. Any disagreements were settled by consensus with a fourth author (MAG-G). The full strategy for identifying potentially relevant studies is detailed in the Supplementary Material S2.

### Study Selection

We included only observational studies that (1) examined the association between dietary copper intake and bone health and (2) measured BMD by DXA as well as other indicative quantitative measures of bone health (bone quantitative ultrasound (QUS) or peripheral quantitative computed tomography (pQCT)). Some authors of the studies included in the meta-analysis were contacted for a request of additional methodological details and data; however, no responses were received. Nonetheless, this lack of supplementary input did not compromise the feasibility of the quantitative synthesis, as the majority of individual studies furnished sufficiently complete and consistent results for incorporation into the overall statistical analysis.

The search results were imported into Rayyan, an online platform facilitating collaborative screening among reviewers [37]. Duplicate records were first eliminated, after which JMM and LMP-P independently assessed all titles and abstracts. Any discrepancies were resolved by consensus, with a third author (MGG-G) consulted when necessary. Both JMM and LMP-P subsequently evaluated the full texts for eligibility. Studies that failed to meet the inclusion criteria were catalogued alongside the corresponding reasons for exclusion. A PRISMA flow diagram was then constructed to illustrate the complete selection process (Supplementary material S3).

### Data Extraction

Two authors (JMM and LMP-P) independently extracted data from the selected studies on a standardized record form. Disagreements were resolved by consensus with a third author (M.A.G.-G.). No studies suitable for meta-analysis of QUS or pQCT outcomes were identified; consequently, only data pertaining to BMD measurements obtained by DXA were extracted and synthesized. The following information was extracted: (1) study population characteristics; (2) WHO region; and (3) BMD measurement site.

### Outcomes

The sole outcome evaluated was the difference in BMD, assessed by DXA at the hip or spine, in relation to dietary copper intake.

### Quality of Evidence

The Newcastle–Ottawa Scale (NOS) [38] was employed to appraise the quality of evidence. This validated instrument, endorsed by the Cochrane Library, comprises eight items that evaluate study selection, comparability and outcomes, yielding a maximum score of nine. The evidence was stratified into three tiers: low quality (< 5 stars), medium quality (5–6 stars) and high quality (> 6 stars). Two reviewers independently evaluated each study. Any discrepancies in risk-of-bias assessments were adjudicated through consultation with a third reviewer. Studies identified as having low quality were nevertheless retained in the analysis (Supplementary Material S4).

### Artificial Intelligence in Manuscript Refinement

Artificial intelligence tools (Grammarly and DeepL) were employed to refine the manuscript’s language, enhance clarity, and improve the communication of complex ideas.

### Statistical Analysis

Review Manager, version 5.2 (The Nordic Cochrane Centre, Copenhagen, Denmark), was used to perform the statistical analyses. A two-sided P value of < 0.05 was considered statistically significant. The data from the eligible articles were categorized into two levels of dietary copper intake: the lowest category, the reference category, and the highest category. A generic inverse-variance random-effects method was used to pool the outcome data for dietary intake of copper categories of the lowest category versus the reference category and the highest category versus the reference category. We used the Q test and I^2^ statistic to evaluate between-study heterogeneity; I^2^ statistics > 50% were considered statistically significant.

## Results

Our initial search identified 692 records. After removing duplicates, two independent reviewers screened the titles and abstracts of the remaining articles. Nineteen full-text articles were then assessed for eligibility. Five studies met the inclusion criteria for the systematic review, specifically Fan et al. [14], Mahdavi-Roshan et al. [7], Chen et al. [29], Pasco et al. [30] and Canal-Macías et al. [28], and four of these studies were incorporated into the meta-analysis [14, 28, 29, 30]. The study by Mahdavi-Roshan et al. [7], although it reports dietary copper intake data and quantitative differences in BMD measured by DXA, did not provide extractable data, and requests for these data from the authors went unanswered.

Our systematic review included five studies with a combined total of 14,396 participants, 14,345 of whom were included in the meta-analysis. The characteristics of the included studies related to the bone health and the copper dietary intake assessment are presented in Table 1.

**Table 1:** Dietary copper intake assessment and BMD measurement methods by WHO region (2015–2025)

Study (year)	WHO Region (WHO classification)	Participants / Setting	*N* (total)	Females *n* (%)	Mean ± SD (or SE) age	BMD measurement sites	Copper dietary intake assesment
Fan et al. [14]	Region of the Americas	General U.S. adults (NHANES 2007-2018)	8 224	4 002 (48.7%)	47.8 ± 0.31 (SE) overall ​	Lumbar spine (L1-4), total femur, femoral neck ​	Two 24-h dietary recalls (mean of both)
Chen et al. [29]	Western Pacific	Hypertensive adults ≥ 60 y, China (NHANES cycles)	5 286	2 425 (45.9%)	69.88 ± 0.13 (SE) overall ​	Total femur, femoral neck (hip) ​	Single 24-h dietary recall
Pasco et al. [30]	Western Pacific	Community women, Australia (Geelong Osteoporosis Study)	522	522 (100%)	Median 52.8 y (IQR 37–66)	Lumbar spine (L2-4), femoral neck, whole body, forearm ​	Semi-quantitative FFQ
Canal-Macías et al. [28]	European Region	Post-menopausal women, Spain	313	313 (100%)	Median 60 y (IQR 6)	Lumbar spine (L2-4), femoral neck; plus pQCT, QUS ​	7-day, 131-item FFQ
Mahdavi-Roshan et al. [7]	Eastern Mediterranean	Osteopenic / osteoporotic post-menopausal women, Iran	51	51 (100%)	58.0 ± 1.2 SD	Femoral neck (T-score–based) ​	3-day food diary

Fan et al. [14] reports only a pooled age (47.0 ± 17.3 y in participants without osteoporosis, 64.9 ± 1.05 y with osteoporosis)

Chen et al. [29] provides the overall mean age with its standard error but no disaggregation by sex.

FFQ: Food-frequency questionnaire

Of the five studies included, two drew upon data from the United States [14, 29], representing the WHO Region of the Americas (AMR). The remaining investigations were conducted in the Islamic Republic of Iran [7], within the WHO Eastern Mediterranean Region (EMR); Australia [30], within the WHO Western Pacific Region (WPR); and Spain [28], which is part of the WHO European Region (EUR). The included investigations employed diverse methodological designs. Four studies were cross-sectional in nature, specifically the analyses conducted by Fan et al. [14], Pasco et al. [30], Canal-Macías et al. [28], and Mahdavi-Roshan et al. [7]. In contrast, Chen et al. [29] utilized a retrospective cohort design. Methodologies across the included studies varied, employing diverse approaches to assess copper intake and bone health outcomes. Fan et al. [14] conducted a cross-sectional analysis utilizing data from the U.S. National Health and Nutrition Examination Survey (NHANES). This involved estimating dietary and supplemental copper intake through two 24-h recalls and measuring BMD using DXA at femur sites and sites on the lumbar spine, with osteoporosis defined by the WHO criteria. Analyses employed logistic and linear regression models adjusted for covariates, accounting for the complex NHANES survey design. Mahdavi-Roshan et al. [7] conducted a cross-sectional study in the Islamic Republic of Iran and compared the mineral status between 51 osteopenic and osteoporotic postmenopausal women. Dietary intake was assessed via a 3-day food recall, and serum copper levels, along with other minerals, were measured using atomic absorption spectrophotometry. Pasco et al. [30] performed a cross-sectional analysis within the population-based Geelong Osteoporosis Study in Australia. Dietary copper intake was assessed via a semiquantitative food frequency questionnaire, and BMD was measured by DXA at multiple skeletal sites. Multivariable regression models were employed to identify associations after adjusting for potential confounders. Canal-Macías et al. [28] presented a cross-sectional analysis of baseline data from a longitudinal study involving 313 postmenopausal women in Spain. Bone health was assessed comprehensively using three distinct quantitative techniques: heel quantitative ultrasound (QUS), DXA at sites on the lumbar spine and hip, and pQCT (peripheral quantitative computed tomography) of the forearm. Dietary copper intake was quantified using a 131-item, 7-day food frequency questionnaire. Statistical analyses included multiple linear regression, both unadjusted and adjusted for age and weight. Finally, Chen et al. [29] used a retrospective cohort study design based on publicly available data from the U.S. NHANES database, with a focus on elderly hypertensive patients. BMD was measured using DXA at femur sites, and dietary intake data, including copper, were utilized. Statistical models included logistic and linear regression, adjusted for various covariates and accounting for the survey design. On the basis of the Newcastle–Ottawa quality assessment scale, the study by Fan et al. [14] achieved the highest score of nine stars. Three studies [28–30] each received eight stars, whereas Mahdavi-Roshan et al. [7] obtained five stars (Supplementary Material S5).

### Findings from the Systematic Review

#### Associations of Dietary Copper Intake with BMD and Osteoporosis Risk

The fundamental conclusions drawn from the five studies regarding the association between copper intake and bone health present a varied picture. The study by Fan et al. [14] concluded that both dietary and total copper intake were positively associated with increased BMD and inversely related to osteoporosis risk in U.S. adults, with higher copper intake corresponding to greater BMD and reduced osteoporosis incidence [14]. Similarly, Chen et al. [29] examined elderly hypertensive participants in the NHANES cohort and reported that increased dietary copper intake was linked to increased lumbar spine and femoral BMD and a decreased likelihood of osteoporosis. Conversely, Mahdavi-Roshan et al. [7] reported no significant differences in copper consumption between postmenopausal women with osteopenia and those with osteoporosis, although the mean intake in both groups remained below the recommended thresholds. In an Australian cross-sectional analysis, Pasco et al. [30] reported that lower dietary copper was independently associated with reduced mean BMD across multiple skeletal sites after adjusting for age and weight. Finally, Canal-Macías et al. [28] assessed postmenopausal Spanish women using QUS, DXA, and pQCT and detected no significant associations between copper intake and bone health parameters following adjustments for age and BMI, a finding they attributed to limited statistical power and a potential type II error.

### Findings from the meta-analysis

#### Dietary Copper Intake and DXA-Derived BMD at the Lumbar Spine

**Fig. 1:**
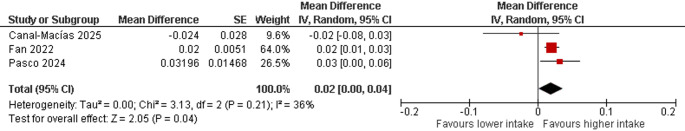
Forest plot of the random-effects meta‐analysis evaluating the associations between higher versus lower dietary copper intake and lumbar spine BMD

A random effects meta-analysis of three studies encompassing 9,059 participants demonstrated that higher dietary copper intake was associated with a modest but statistically significant increase in lumbar spine BMD (MD 0.02 g/cm^2^; 95% CI 0.00–0.04; *p* = 0.04) (Fig. 1). Between-study heterogeneity was moderate and not statistically significant (I^2^ = 36%; χ^2^ = 3.13, df = 2; *p* = 0.21; τ^2^ = 0), suggesting that the observed effect is reasonably consistent across studies. These findings indicate a potential beneficial effect of greater copper consumption on lumbar spine bone density. A formal assessment of publication bias (e.g., Egger’s test) is not recommended with fewer than ten studies, precluding a robust evaluation of small-study effects.

### Dietary Copper Intake and DXA-Derived BMD at the Hip

**Fig. 2:**
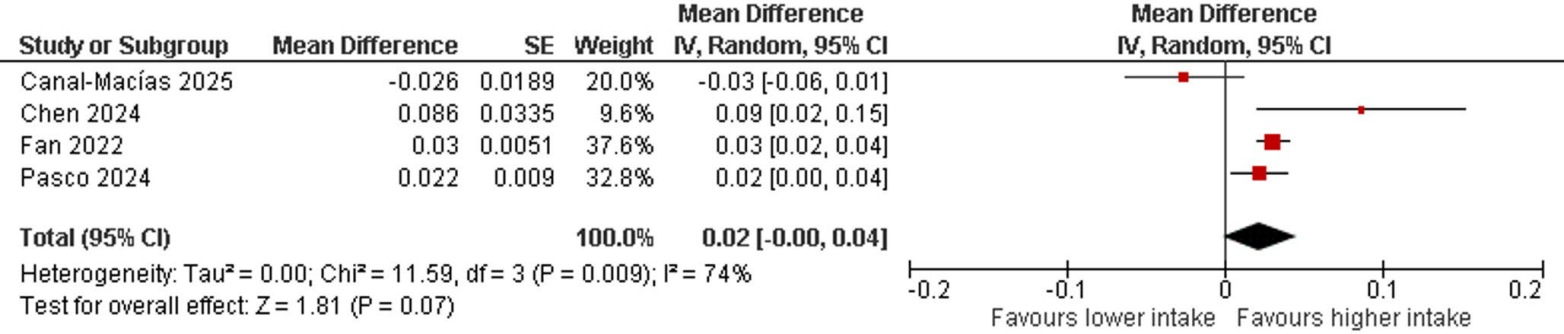
Forest plot of a random-effects meta-analysis of four studies evaluating the association between dietary copper intake and hip bone mineral density (DXA)

A random-effects meta‐analysis of four studies [14, 28, 29, 30] comprising a total of 14,345 participants evaluated the associations between higher versus lower dietary copper intake and hip BMD measured by DXA. The pooled estimate indicated a nonsignificant mean difference of 0.02 g/cm^2^ (95% CI − 0.00 to 0.04; *p* = 0.07) (Fig. 2), suggesting a trend towards higher hip BMD with greater copper intake that did not reach statistical significance. Between‐study heterogeneity was substantial and statistically significant (I^2^ = 74%; χ^2^ = 11.59, df = 3; *p* = 0.009; τ^2^ = 0), reflecting considerable variability in effect estimates across the included studies. Formal assessment of publication bias was not undertaken owing to the limited number of studies available for meta-analysis.

## Discussion

To our knowledge, this is the first systematic review and meta-analysis examining the effects of dietary copper intake on bone health as determined by DXA. Our pooled analysis suggested a modest positive association between higher copper consumption and increased BMD. However, these findings must be interpreted with caution given the limited number of studies included and their exclusively observational designs, which limit the reliability and generalizability of the results.

Copper plays fundamental biological roles in overall bone health, especially in the bone tissue that composes the hip and spine. Copper is an essential trace element that cannot be synthesized by the human body and must be obtained through the diet for the maintenance of physiological health [3, 4]. A key role of copper in bone health is in the cross-linking of collagen and elastin via the enzyme lysyl oxidase [39, 40]. Collagen is a major component of the bone extracellular matrix, and the integrity of this matrix is very important for the strength and plastic deformation of the bone [14, 20]. In addition, copper has a positive effect on osteoblast proliferation and function and indirectly promotes osteogenic differentiation of bone marrow mesenchymal stem cells [41]. Copper deficiency, therefore, can reduce bone formation [14]. These plausible mechanisms suggest that adequate copper intake may positively influence bone density and strength at sites such as the hip and spine.

In our study, we observed that high copper intake is associated with significantly higher BMD in the lumbar spine, which suggests that this micronutrient promotes bone health through its involvement in either bone morphology or bone formation. These findings are consistent with those of Fan et al. [14], who reported that higher levels of copper intake are associated with increased total BMD of the spine, especially in women; likewise, Pasco et al. [30] reported that lower dietary intake of copper is associated with reduced BMD values at different skeletal sites, including the spine, independent of height. Pasco et al. [30] reported that the lowest quartile of dietary copper intake (< 0.86 mg/day) and the highest quartile (≥ 1.51 mg/day) had a relatively high mean total column BMD of 0.02 g/cm^2^ in the studied women. Similarly, in the study by Pasco et al. [30], low dietary intake of copper (< 1.26 mg/day) was consistently associated with lower BMD; however, in fully adjusted models (including age, weight and height), small but statistically significant differences were identified. In contrast, Canal-Macías et al. [28], who evaluated lumbar BMD by DXA in Spanish postmenopausal women, reported no association between copper intake and bone parameters, a discrepancy that could be explained by a limited sample size or a possible type II error compared with larger studies. The pooled analysis of these results, through a meta-analysis, helps to alleviate the limitations of the statistical power of each individual investigation and provides evidence that copper positively effects lumbar BMD; however, this effect appears modest and is based on a small number of studies and pooled effects. Taken together, our data provide additional evidence of the potential benefit of adequate copper intake on lumbar bone density, although prospective and more statistically powered research is needed to confirm this relationship fully.

Several studies have investigated this relationship specifically at the hip/femoral neck, with results varying among sources. A study by Fan et al. [14], which used data from U.S. adults, revealed that dietary and total copper intake was positively associated with increased BMD at several bone sites, including the whole femur. Specifically, the mean whole femur BMD of the highest quartile of dietary copper intake (≥ 1.51 mg/day) was 0.03 g/cm^2^ greater than that of the lowest quartile. This positive association with whole femur BMD was significant in both males and females in the sex-stratified analysis [14]. A study by Pasco et al. [30] in women revealed that low dietary intake of copper was consistently associated with lower BMD at multiple skeletal sites, including the femoral neck [30]. In fully adjusted models, small but statistically significant differences in BMD were identified; for low copper intake, the mean BMD was 3.3% lower in the femoral neck (*p* = 0.008). In elderly hypertensive patients, dietary intake of copper (specifically, intake ≥ the recommended daily amount) was positively correlated with BMD in the whole femur and femoral neck after adjusting for covariates [29]. This intake ≥ the recommended daily intake (RDA) was associated with a β of 0.086 (95% CI 0.021–0.152) for the whole femur and 0.097 (95% CI 0.016–0.178) for the femoral neck. The authors conclude that higher copper intake may contribute to improved bone health and increased BMD, a finding consistent with the study by Fan et al. [14]. On the other hand, our study [28] in Spanish postmenopausal women, which used DXA to measure BMD in the femoral neck and femoral trochanter, did not find statistically significant associations between dietary copper intake and BMD in these regions after adjusting for age and weight. In this case, and in view of the previous results available in the literature, the absence of significant findings could be due to a possible limitation of statistical power (a type II error), given our relatively smaller sample size compared with studies that did find significant associations. This problem should be partially solved by analysing the results of all the studies included in the meta-analysis. However, in this case, the pooled analysis did not show a positive association between copper intake and hip BMD, indicating that copper did not influence hip BMD. The results obtained from the individual results could involve a type I error due to the intrinsic nature of observational studies. The presence of biases in the analysis with respect to dietary copper intake could alter the result so that a positive association was found between dietary intake and hip BMD in the individual studies, but this result was not found in the pooled meta-analysis.

Studying the association between copper intake and bone health presents significant challenges due to the diversity of copper intake measurement methods and the different endpoints used among the studies [28]. Methods of assessing dietary intake vary, including the use of 24-hour recall surveys (averages of two [14] or based on a single interview [29]) and FFQs, some of which are detailed and semiquantitative [30] while others are more complete [28]. These methods differ in their ability to capture long-term versus recent habitual intake [7, 14, 28, 29], and both are subject to recall bias and inaccuracies based on standard nutritional composition databases [28, 30]. In addition, some studies included the intake of copper supplements [14, 30]. With respect to benchmarks, some studies categorized copper intake into quartiles or tertiles on the basis of their specific population distribution [14, 28, 30], which means that the absolute ranges of intake in each category differ considerably across studies, making direct comparisons of findings difficult. Other studies have used fixed thresholds based on the recommended daily intake (RDA) values or other reference values [29, 30], which introduces variability if these values differ by region or do not fully capture the complex relationship between intake and bone health. This lack of standardization in measurement and categorization, coupled with the heterogeneity of the study populations and bone health metrics employed, limits the direct ability to compare results and may contribute to the discrepancies observed.

In the present study, we recognize the following limitations. The studies included in the meta-analysis used a variety of methods, such as 24-hour recall surveys and FFQs. These methods are inherently susceptible to recall bias, which can lead to inaccuracies in the estimation of long-term habitual intake. In addition, the use of standard nutritional composition databases with these questionnaires can lead to discrepancies between estimated and actual intakes, as they do not always reflect individual or geographic variations in the nutrient content of foods. The difficulty in capturing total copper intake, including nondietary sources such as water, is another limitation in accurately assessing exposure. Another important limitation is the cross-sectional nature of several studies included in the meta-analysis. This design precludes establishing temporal and causal relationships between copper intake and bone health outcomes, as exposure and outcome are measured simultaneously. Although different studies adjust for multiple covariates, there is the possibility of residual confounding effects due to unmeasured factors that could influence both copper intake and bone health. This can lead to biased associations if all relevant factors are not fully controlled for. Some studies also noted that adjustments for age and weight might not adequately control for confounding factors [30]. Finally, the heterogeneity of the populations studied (in terms of age, menopausal status, geographic origin and possible health conditions such as hypertension) also limits the generalizability of the results.

## Conclusion

In conclusion, this meta-analysis revealed a positive, albeit modest, association between habitual copper intake and bone health indices measured by DXA, especially in the lumbar spine. However, the high heterogeneity (derived from the different methodologies for quantifying copper intake, the variability of the populations studied and the different study designs) limits the interpretation of the magnitude of the effect. Given that the effect is modest and that methodological discrepancies preclude establishing a firm causal relationship, the implementation of specific public health dietary recommendations to increase copper intake for the prevention of osteoporosis is not warranted at this time. Prospective studies with standardized intake measurements and longer follow-up periods are necessary to confirm this association and assess its clinical relevance.

## Supplementary Information

Below is the link to the electronic supplementary material.

Supplementary File S1. PRISMA 2020 checklist

Supplementary File S2. Search Strategies

Supplementary File S3. Flow diagram

Supplementary File S4. Newcastle-Ottawa Quality Assessment Scale

Supplementary File S5 Application of NO-Scale

## Data Availability

All data used in this meta-analysis can be retrieved from the original sources by accessing the databases specified in the methodology section. The study is fully reproducible in this regard.
