# Supplementary material for: Dietary Copper Intake and Bone Health: A Systematic Review and Meta-Analysis of Observational Studies
Source: Calcif Tissue Int. 2025 Dec 9;116(1):149. doi: 10.1007/s00223-025-01463-w (PMC12686089; doi:10.1007/s00223-025-01463-w)
Supplement: Supplementary file 2 — Supplementary File S2. Search Strategies [file 223_2025_1463_MOESM2_ESM.pdf]

**SCOPUS**

(TITLE-ABS-KEY("copper") OR TITLE-ABS-KEY("coppers") OR TITLE-ABS-KEY("copper s"))

AND

(TITLE-ABS-KEY("eating") OR TITLE-ABS-KEY("dietary intake") OR (TITLE-ABS-KEY("dietary") AND TITLE-ABS-KEY("intake")))

AND

((TITLE-ABS-KEY("bone and bones") OR TITLE-ABS-KEY("bone") OR (TITLE-ABS-KEY("bone") AND TITLE-ABS-KEY("bones"))

AND

(TITLE-ABS-KEY("health") OR TITLE-ABS-KEY("health s") OR TITLE-ABS-KEY("healthful") OR TITLE-ABS-KEY("healthfulness") OR TITLE-ABS-KEY("healths")))

OR

(TITLE-ABS-KEY("osteoporosis") OR TITLE-ABS-KEY("osteoporoses") OR TITLE-ABS-KEY("postmenopausal osteoporosis") OR (TITLE-ABS-KEY("osteoporosis") AND TITLE-ABS-KEY("postmenopausal")))

OR

(TITLE-ABS-KEY("bone density") OR (TITLE-ABS-KEY("bone") AND TITLE-ABS-KEY("density")) OR TITLE-ABS-KEY("bone mineral density") OR (TITLE-ABS-KEY("bone") AND TITLE-ABS-KEY("mineral") AND TITLE-ABS-KEY("density"))))

**OVID MEDLINE**

1. exp Copper/ or copper.mp. or coppers.mp. or "copper s".mp.

2. exp Eating/ or eating.mp. or (dietary.mp. and intake.mp.) or "dietary intake".mp.

3. exp Bone and Bones/ or (bone.mp. and bones.mp.) or "bone and bones".mp. or bone.mp.

4. exp Health/ or health.mp. or "health s".mp. or healthful.mp. or healthfulness.mp. or healths.mp.

5. 3 and 4

6. exp Osteoporosis/ or osteoporosis.mp. or osteoporoses.mp. or exp Osteoporosis, Postmenopausal/ or (osteoporosis.mp. and postmenopausal.mp.) or "postmenopausal osteoporosis".mp.

7. exp Bone Density/ or (bone.mp. and density.mp.) or "bone density".mp. or (bone.mp. and mineral.mp. and density.mp.) or "bone mineral density".mp.

8. 5 or 6 or 7

9. 1 and 2 and 8

**WEB OF SCIENCE**

TS=("copper" OR "coppers" OR "copper s")

AND

TS=("eating" OR ("dietary" AND "intake") OR "dietary intake")

AND

(

(

TS=("bone and bones" OR ("bone" AND "bones") OR "bone")

AND

TS=("health" OR "health s" OR "healthful" OR "healthfulness" OR "healths")

)

OR

TS=("osteoporosis" OR "osteoporoses" OR "osteoporosis, postmenopausal" OR ("osteoporosis" AND "postmenopausal"))

OR

TS=("bone density" OR ("bone" AND "density") OR ("bone" AND "mineral" AND "density") OR "bone mineral density")

)

**PUBMED MEDLINE**

("copper"[MeSH Terms] OR "copper"[All Fields] OR "coppers"[All Fields] OR "copper s"[All

Fields]) AND ("eating"[MeSH Terms] OR "eating"[All Fields] OR ("dietary"[All Fields] AND

"intake"[All Fields]) OR "dietary intake"[All Fields]) AND ((("bone and bones"[MeSH Terms]

OR ("bone"[All Fields] AND "bones"[All Fields]) OR "bone and bones"[All Fields] OR

"bone"[All Fields]) AND ("health"[MeSH Terms] OR "health"[All Fields] OR "health s"[All

Fields] OR "healthful"[All Fields] OR "healthfulness"[All Fields] OR "healths"[All Fields])) OR

("osteoporosis"[MeSH Terms] OR "osteoporosis"[All Fields] OR "osteoporoses"[All Fields]

OR "osteoporosis, postmenopausal"[MeSH Terms] OR ("osteoporosis"[All Fields] AND

"postmenopausal"[All Fields]) OR "postmenopausal osteoporosis"[All Fields]) OR ("bone

density"[MeSH Terms] OR ("bone"[All Fields] AND "density"[All Fields]) OR "bone

density"[All Fields] OR ("bone"[All Fields] AND "mineral"[All Fields] AND "density"[All Fields])

OR "bone mineral density"[All Fields]))

**EMBASE**

('copper'/exp OR 'copper':ab,ti OR 'coppers':ab,ti OR 'copper s':ab,ti) AND ('eating'/exp OR 'eating':ab,ti OR ('dietary':ab,ti AND 'intake':ab,ti) OR 'dietary intake':ab,ti) AND ((('bone'/exp OR 'bone and bones'/exp OR 'bone':ab,ti OR 'bones':ab,ti OR 'bone and bones':ab,ti) AND ('health'/exp OR 'health':ab,ti OR 'health s':ab,ti OR 'healthful':ab,ti OR 'healthfulness':ab,ti OR 'healths':ab,ti)) OR ('osteoporosis'/exp OR 'osteoporosis':ab,ti OR 'osteoporoses':ab,ti OR 'postmenopausal osteoporosis'/exp OR ('osteoporosis':ab,ti AND 'postmenopausal':ab,ti) OR 'postmenopausal osteoporosis':ab,ti) OR ('bone density'/exp OR 'bone density':ab,ti OR ('bone':ab,ti AND 'density':ab,ti) OR 'bone mineral density'/exp OR ('bone':ab,ti AND 'mineral':ab,ti AND 'density':ab,ti) OR 'bone mineral density':ab,ti))
