# Supplementary figures and images for: Dietary Copper Intake and Bone Health: A Systematic Review and Meta-Analysis of Observational Studies
Source: Calcif Tissue Int. 2025 Dec 9;116(1):149. doi: 10.1007/s00223-025-01463-w (PMC12686089; doi:10.1007/s00223-025-01463-w)

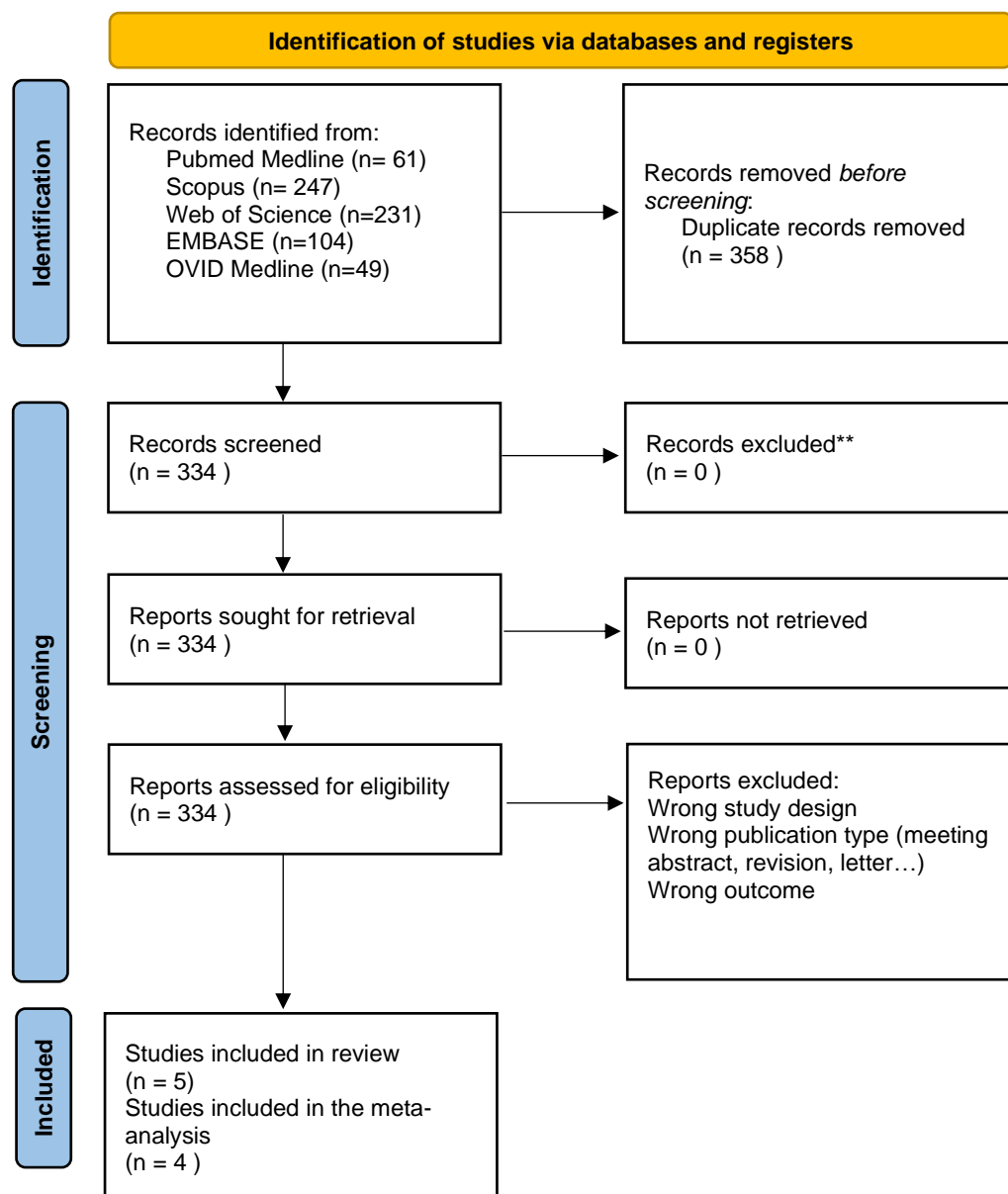

Supplement: Supplementary file 3 — Supplementary File S3. Flow diagram [file 223_2025_1463_MOESM3_ESM.pdf]
