# Supplementary material for: Dietary Copper Intake and Bone Health: A Systematic Review and Meta-Analysis of Observational Studies
Source: Calcif Tissue Int. 2025 Dec 9;116(1):149. doi: 10.1007/s00223-025-01463-w (PMC12686089; doi:10.1007/s00223-025-01463-w)
Supplement: Supplementary file 5 — Supplementary File S5 Application of NO-Scale [file 223_2025_1463_MOESM5_ESM.pdf]

1. Quality Assessment Table for Case-Control Study (Fan et al., 2022)

NOS Category	Criteria	Score	Range of Score	Justification
Selection	Case definition adequate	★	★	Osteoporosis defined based on BMD ≥ 2.5 SD below mean; uses standardized WHO criteria
	Representativeness of the cases	★	★	Cases were drawn from NHANES
	Selection of controls	★	★	Controls were selected from the same NHANES cohort
	Definition of controls	★	★	Controls were free from osteoporosis based on defined BMD criteria.
Comparability	Comparability of cases and controls on confounders	★★	★★	Multivariable models adjusted for major confounders (age, gender, race, BMI, socioeconomic and health status factors…
Exposure	Ascertainment of exposure	★	★★	Copper intake data was collected using validated 24-hour dietary recalls and supplement use assessments.
	Same method of ascertainment for cases and controls	★	★	Both cases and controls underwent identical NHANES dietary data collection protocols.
	Non-response rate	★	★	Exclusions were documented; included only participants with complete data for BMD, copper intake, and covariates.

Total Score: 9 out of 9 stars

2.- Newcastle-Ottawa Quality Assessment Scale (Modified for Cross-Sectional Studies) (Pasco et al., 2024)

Domain	Item	Score	Justification
**Selection**	1. Representativeness of the sample	★	The sample is drawn randomly from the Geelong Osteoporosis Study
	2. Non-respondents	★	The response rate was 77%. Participants with incomplete data were excluded.

Domain	Item	Score	Justification
	3. Ascertainment of the exposure (risk factor)	★	A validated semi-quantitative food frequency questionnaire was used
**Comparability**	1. Control for confounding factors	★★	Models adjusted for age, weight, height, calcium intake, energy intake, physical activity, hormone therapy, smoking, alcohol, and glucocorticoids.
**Outcome**	1. Assessment of the outcome	★	Bone mineral density (BMD) was measured using DXA.
	2. Statistical test	★	Appropriate statistical tests were used (ANCOVA, multivariable regression),

Total Score: 8 out of 8 stars

3.- Newcastle-Ottawa Quality Assessment Scale (Adapted for Cross-Sectional Studies) (Canal-Macías et al., 2025)

Domain	Item	Score	Justification
**Selection**	1. Representativeness of the sample	★	The sample consisted of 313 community-dwelling postmenopausal women from a larger longitudinal study.
	2. Non-respondents	★	A 100% response rate was reported for the dietary questionnaire.
	3. Ascertainment of the exposure (risk factor)	★	Copper intake was assessed using a validated 131-item food frequency questionnaire.
**Comparability**	1. Control for confounding factors	★★	Statistical models adjusted for key confounders
**Outcome**	1. Assessment of the outcome	★	Bone health was measured using three standardized, validated techniques (DXA, QUS, pQCT).
	2. Statistical test	★	Appropriate statistical tests (multiple linear regression, nonparametric comparisons) were used.

Total Score: 8 out of 8 stars

4.- **Newcastle-Ottawa Quality Assessment Scale (Adapted for Cross-Sectional Studies) (Chen et al., 2024)**

**Domain**	**Item**	**Score**	**Justification**
**Selection**	1. Representativeness of the sample	★	The sample was extracted from NHANES, specifically elderly hypertensive adults.
	2. Non-respondents	★	The study transparently handled exclusions and documented complete case inclusion.
	3. Ascertainment of the exposure (risk factor)	★	Dietary intake was measured using validated 24-hour dietary recalls by trained interviewers.
**Comparability**	1. Control for confounding factors	★★	The analysis adjusted for a wide array of covariates: demographic, socioeconomic, health-related, and dietary factors (age, BMI, comorbidities, energy intake…).
**Outcome**	1. Assessment of the outcome	★	Bone mineral density was assessed using DXA.
	2. Statistical test	★	Statistical methods were appropriate (logistic and linear regression with weighting)

Total Score: 8 out of 8 stars

NOTE: *Although the study is labeled as a retrospective cohort, it utilizes cross-sectional data from NHANES where both exposure (dietary intake) and outcome (osteopenia/osteoporosis) are measured simultaneously, without longitudinal follow-up. Therefore, we have considered the modified Newcastle-Ottawa Scale for cross-sectional studies to be a more methodologically appropriate tool for quality assessment.*

5.- Newcastle-Ottawa Quality Assessment Scale (Adapted for Cross-Sectional Studies) (Mahdavi-Roshan et al., 2015)

**Domain**	**Item**	**Score**	**Justification**
**Selection**	1. Representativeness of the sample	★	The sample was randomly selected from a rheumatology clinic; although not population-wide, it is reasonably representative of postmenopausal women at clinical risk.
	2. Non-respondents	x	No details are provided about response rates or comparisons with non-respondents
	3. Ascertainment of the exposure (risk factor)	★	Dietary intake was measured using 3-day food recalls checked by a nutritionist
**Comparability**	1. Control for confounding factors	x	No multivariate analysis or adjustments for confounders like age or BMI were reported.

Domain	Item	Score	Justification
**Outcome**	1. Assessment of the outcome	★	BMD was assessed using DXA.
	2. Statistical test	★	Statistical tests were appropriate (t-tests, correlations

Final Score: 5 out of 8 stars
